# Bionanotechnology: Silver Nanoparticles Supported on Bovine Bone Powder Used as Bactericide

**DOI:** 10.3390/ma13020462

**Published:** 2020-01-18

**Authors:** Sergio Arturo Gama-Lara, Martha Stephanie Pérez Mendoza, Alfredo Rafael Vilchis-Nestor, Reyna Natividad

**Affiliations:** 1 Engineering department, campus Toluca, Universidad del Valle de México, De Las Palmas 439, San Jorge Pueblo Nuevo, Metepec 52164, Mexico; gamaliel350@hotmail.com; 2 Centro Conjunto de Investigación en Química Sustentable UAEM-UNAM, Universidad Autónoma del Estado de México, Carretera Toluca-Atlacomulco Km 14.5, San Cayetano, Toluca 50200, Mexico; arvilchisn@uaemex.mx

**Keywords:** nanocomposite, *Escherichia coli*, bactericide effect, Ag nanoparticles, minimum inhibitory concentration

## Abstract

Bionanotechnology is a relatively new term that implies the use of some biological material or organisms in order to prepare nanosystems or nanoparticles. This work presents the preparation and bactericide application of a sustainable nanometric system (silver nanoparticles) using a waste biological support (bovine bone powder). This system was prepared by the method of metallic salt reduction, using NaBH_4_ as reducing agent and AgNO_3_ as metallic salt. Two silver contents were analyzed, 1% and 5% weight. The latter was found to be more efficient than the former. Transmission electronic microscopy shows an average size of 10.5 ± 3.3 nm and quasi-sphere morphology. The antimicrobial assay shows that a 5% weight content of silver had a bactericide effect for *Escherichia coli* at 46.8 min of exposure. The minimum inhibitory concentration (MIC) of silver nanoparticles supported on bovine bone powder for *Escherichia coli* was 7.5 µg/mL. The biocomposite exhibits a specific antibacterial kinetics constant (k) of 0.1128 min^−1^ and decimal reduction time (DRT) of 20.39 min for *Escherichia coli*. Thus, it was concluded that a biocomposite was prepared with a biodegradable, waste, and low-cost support, under mild conditions (room temperature and atmospheric pressure) and using water as solvent.

## 1. Introduction

Nanometric systems have been the subject of study of a large number of articles in the last decades. This is because nanostructured systems have better chemical, mechanical, and catalytic properties than bulk materials and can be applied in various technologies. On the other hand, these systems are more efficient and less expensive than their counterparts, the bulk materials [1,2,3,4,5,6,7,8]. Due to the unique properties of nanometric systems, the number of their applications is vast. In this sense, the objective of this work was to look for a sustainable bactericide system, environmentally friendly, with a natural support, that could be synthesized under room temperature, atmospheric pressure, and using water as a solvent. To achieve so, silver nanoparticles were synthesized on bovine powder and the resulting system was tested in the elimination of a Gram negative bacterium such as *Escherichia coli*. This organism was proposed because it is one of the most difficult to treat, being the cause of severe diarrhea due to consumption of water contaminated with coliforms. This type of bacteria has become multi-resistant to antibiotics, making its treatment rather difficult and is an important cause of death in places where people do not have access to drinking water [4,9,10,11,12,13,14,15,16,17].

An important strategy to limit the size, protect, and recover the synthesized nanoparticles, is the support adequate selection. In this sense, various supports have been assessed: (a) of biological origin like core/shell [18,19], cotton [20,21], wood [3], or natural fibers [22], and (b) man-made such as titania [23], various polymers [24,25,26,27], graphene sheets [28], or ceramics [29]. Regarding bovine bone powder, it has been shown to exhibit a good mechanical, thermal, and chemical resistance (it does not oxidize) and also shows great affinity to metallic nanoparticles because of hydroxy (OH^−^) and phosphate (PO_4_^3−^) groups, which exhibit a rather high ability to anchor the nanoparticles to the support. The bone is composed mostly by hydroxyapatite (HPA). Besides, it is a waste material, renewable, biodegradable, easily obtained, and treated. Thus, these characteristics made out of bovine powder a very attractive material to be used as support of nanoparticles, specifically speaking of silver nanoparticles. It is expected that silver nanoparticles improve the number of active sites that attract the bacteria membrane thus inhibiting their reproduction.

Recently, an increasing number of articles have been published concerning the properties of silver nanoparticles. Their bactericidal effect, for instance, has been a very recurring topic. Such articles have shown that nanometric silver eliminates a wide range of bacteria and has a promising large number of applications in medicine and industry [17,30,31].

In this work, the synthesis method for silver nanoparticles was the reduction of metal salts, using sodium borohydride (NaBH_4_) as a reducing agent and AgNO_3_ as the metal salt. Through this method the size, shape, and dispersion of the synthesized particles can be controlled by varying the concentration of the metal salt or the reduction time [32,33]. The characterization was carried out by means of transmission electron microscopy (TEM) through which the size, shape, polydispersity of the metal nanoparticles, the interplanar distance of the atoms, as well as the support where they are found can be observed.

## 2. Experimental

### 2.1. Bovine Bone Powder Preparation

The bovine bone was purchased from a local grocery store (Plan de Agua Prieta, La Magdalena, 50190, Toluca, Edo. de Mexico, Mexico). The bovine bone femur was washed in boiled water for 30 min and cleaned, later the piece was cut into small pieces. These pieces were then finely ground and for this purpose a moto-tool with tungsten rotating piece was used; then the powder was sieved with a 150 mesh.

### 2.2. Synthesis

The metallic salt reduction method was used for the synthesis of Ag NP’s. AgNO_3_ was used as metallic salt and NaBH_4_ was the reducing agent. Two solutions (solution 1 and solution 2) were prepared. For solution 1, 8.495 mg of AgNO_3_ were dissolved in 50 mL of deionized water (concentration = 0.001 M). For solution 2, 1.89 mg of NaBH_4_ were dissolved in 50 mL of water (concentration = 0.001 M), pH was not adjusted at any time. Two silver/bovine bone powder systems with different silver content were prepared. The first one with 1% weight of silver and the second one with 5%. For the first content (1%), an amount of 539.35 mg of bovine bone powder was immersed in 50 mL of solution 1 during 30 s and then filtered. For the second content (5%), 107.87 mg of bovine bone powder was immersed into solution 1. The reduction of Ag (I) ions was carried out with solution 2 during 30 min, at room temperature and atmospheric pressure. The resulting powder was then filtered under vacuum and dried overnight at room temperature. It is worth noticing that by this method, the typical calcination step is eliminated. The reaction can be written as follows [34],

$$8Ag^{+}+BH_{4}{}^{-}+4H_{2}O\;\to 8\mathrm{Ag}+B{\left(OH\right)}_{4}{}^{-}+8H^{+}.$$

### 2.3. Characterization

Transmission electronic microscopy (TEM) studies were performed in a JEOL microscope (JEOL, Akishima, Tokyo, Japan) JEM-2100 operated at 120 kV of accelerating voltage. The silver-impregnated bovine bone powders were suspended in 2-propanol and then ultrasonically dispersed for 5 h at room temperature. A drop of this suspension was then placed on a Cu-grid coated with a holey carbon film.

### 2.4. Antimicrobial Assay

An *Escherichia coli* O157:H7 strain from institution strain repository was used. This was reseeded in MacConkey agar. A dilution of 1.164 × 10^5^ CFU in 10 mL of distilled water was prepared in three different test tubes, tube 1 received no treatment, tube 2 was added with 1.5 mg of the support at 1%, and tube 3 was added with 1.5 mg of the system with 5% silver content. The tubes were incubated at 37 °C during 24 h. After this time, the petri dish with MacConkey agar was divided into three parts to make the reseeding, then an aliquot of 7 micro liters of each tube was taken and reseeded as follows: vial 1 was used for control, vial 2 was prepared with the 1% Ag/bone system, and vial 3 was prepared with the 5% Ag/bone system. This was conducted in order to compare the effect of Ag content on the bactericidal effectiveness of the prepared system, and to establish the minimum inhibitory concentration (MIC). Then all vials were incubated for 24 h at 37 °C.

Once the best Ag/bovine bone powder system (5%) was established, a further experiment was conducted in order to establish the bacteria decay kinetics. Then a new solution was made of 1.164 × 10^5^ CFU in 10 mL of distilled water in a test tube. The petri dish with MacConkey agar was divided in two, in order to make the new reseeded. An aliquot of 10 micro liters was taken, and reseeded into the petri dish. This process was conducted at different times, the first aliquot was the control, the second aliquot was 5 min after adding the prepared system, and the next aliquots were each 10 min until 65 min were reached.

## 3. Results and Discussion

### 3.1. Characterization

**Figure 1:**
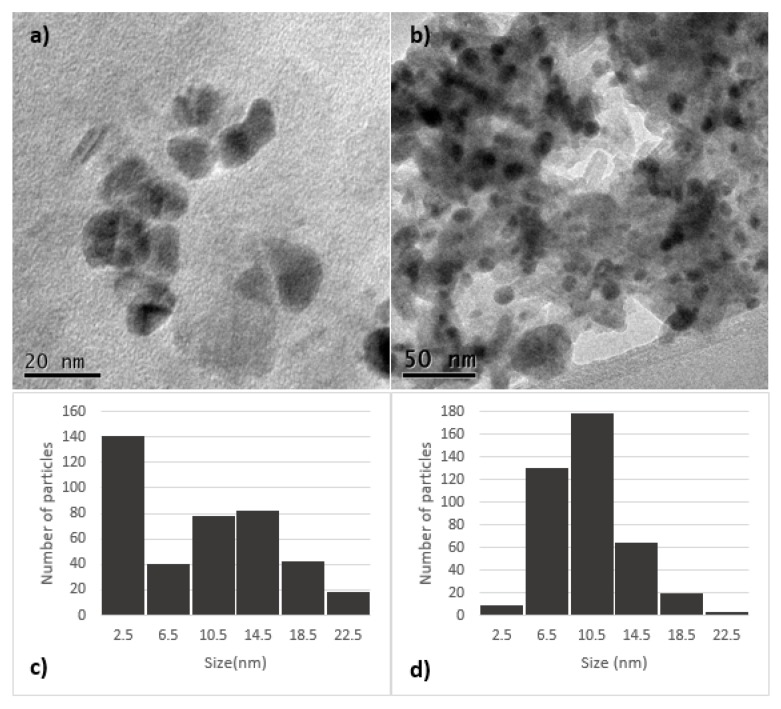
(**a**) TEM image of 1% Ag/bovine bone powder, (**b**) TEM image of 5% Ag/bovine bone powder, (**c**) size distribution of silver nanoparticles (1%) on bovine powder, and (**d**) size distribution of silver nanoparticles (5%) on bovine powder.

Figure 1c shows the particle size distribution in the 1% Ag/bovine bone powder. It can be observed that the distribution is not normal and therefore it would not be accurate to provide an average size. It can be observed, however, that the size of about a third part of the whole accounted particles was 2.5 nm while the rest exhibited a diameter that ranges between 6.5 and 22.5 nm. Regarding the material with 5% of Ag NP’s (Figure 1d), the particles exhibited an average size of 10.5 ± 3.3 nm. To conduct this analysis, the open access software ImageJ was used. An example of the process is given as Supplementary Information. More TEM images of the 5% Ag/bone powder are depicted in Figures S1 and S2. Figure 1b shows silver nanoparticles (5%) supported on the bovine bone powder with better polydispersity and homogeneity than the observed in the 1% Ag system (Figure 1a). Increasing silver content on bovine bone powder from 1% to 5%, leads to a higher amount of nanoparticles with an average size of 10.5 nm and size homogeneity is also improved. These images correspond to a randomly chosen part of the substrate, and can be considered as representative of the overall size and shapes of the particles.

**Figure 2:**
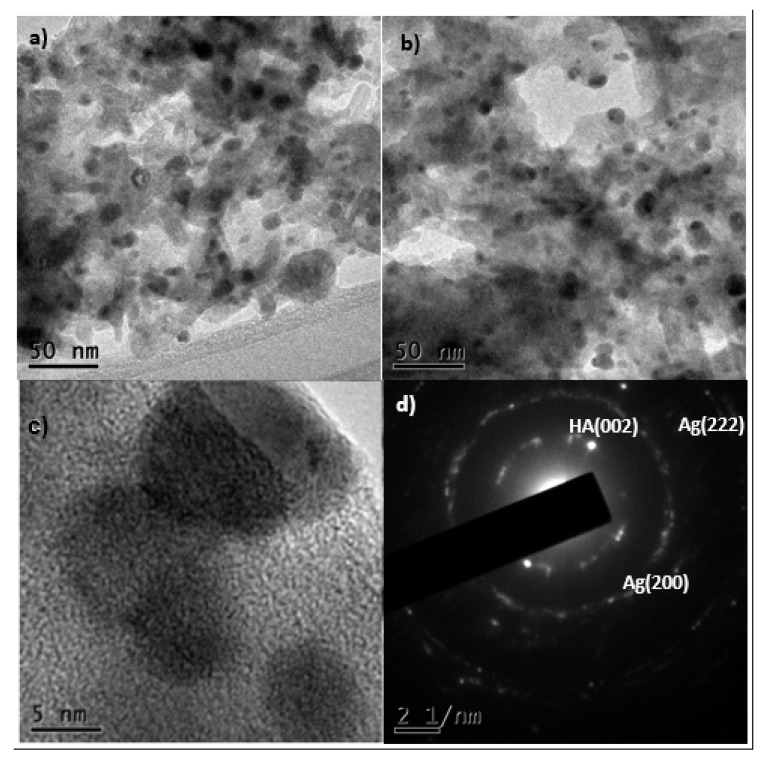
(**a**) and (**b**) TEM images of Ag nanoparticles (5%), (**c**) HRTEM, and (**d**) selected area electron diffraction (SAED) images of Ag nanoparticles in bovine bone powder (5%), in different magnifications 120 kV.

Figure 2 shows micrographs at different magnifications. In these images the silver nanoparticles could be observed darker due to their higher atomic number, and they were supported on the bovine bone powder. The shape of the nanoparticles is quasi-spherical with good polydispersity. The system remained bound due to the electrostatic attraction between the silver and the groups hydroxy and phosphate of the support. Figure 2d corresponds to a selected area electron diffraction (SAED) image that shows spots corresponding to (200) and (222) [35] reflections of Ag NPs lattice planes, which confirms the FCC lattice and thus indicates polycrystalline Ag NPs. As well, we found the diffraction pattern of the hydroxyapatite in the direction (002) [36], which shows that even after the sonication process the silver nanoparticles were still attached to the hydroxyapatite, showing a strong link between them.

Kim et al. [37] and Suber et al. [38] have suggested that a bimodal mechanism regulate the growth and symmetry of the nanoparticles. The results obtained regarding crystalline planes, observed in the selected area electron diffraction (SAED), of the nanoparticles, suggest that a fast reduction mechanism regulate the synthesis of silver nanoparticles with NaBH_4_ as a reduction agent. Otherwise, the porosity of the bovine bone powder does not allow the agglomeration, getting a rather small particle size (average = 10 nm with the 5% Ag/bone system).

**Figure 3:**
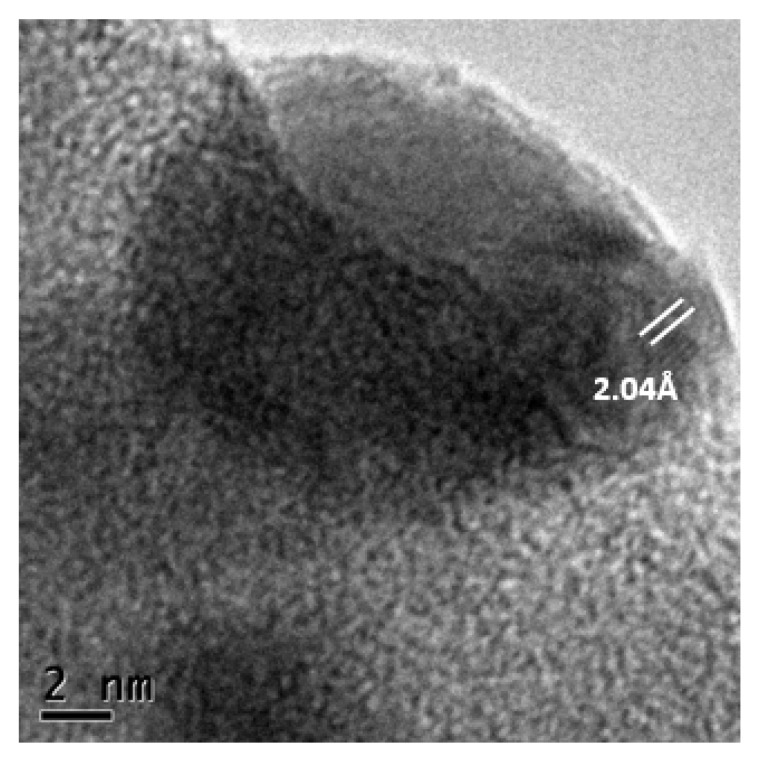
HRTEM micrograph of Ag-nanobiocomposite 5% Ag/bovine bone powder.

Figure 3 shows 2.04 Å lattice planes, corresponding to the (200) plane of cubic Ag [35]. Confirming the information obtained in the SAED of Figure 2d.

### 3.2. Antibacterial Action

**Figure 4:**
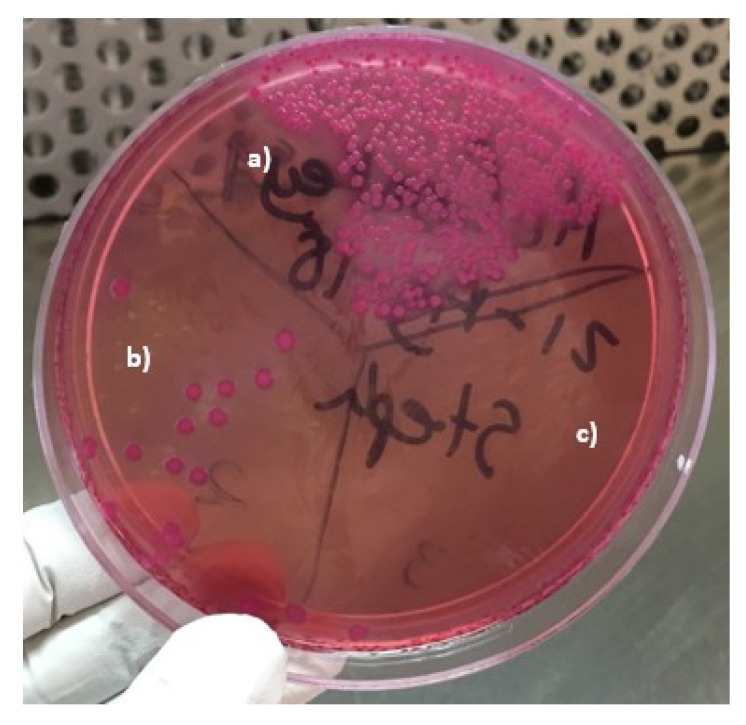
Photograph of plate with *Escherichia coli* and MacConkey agar showing the comparison of bactericidal properties. (**a**) Sample without Ag/bone system, (**b**) sample with 1% Ag/bone system, and (**c**) sample with 5% Ag/bone system.

Figure 4 shows the bacteria growth of the *Escherichia coli* strain. Figure 4a received no treatment and 348 CFU were counted. The section shown in Figure 4b was added with 1.5 mg of the 1% Ag/bone system and 18 CFU were counted. The section shown in Figure 4c was added with 1.5 mg of 5% Ag/bone system and a very effective bactericidal effect was demonstrated by not finding any CFU. The bactericidal effect of silver was expected, and is due to the negative charges of the phosphate groups of the bacterial nucleotides being attracted by the positive charges of silver, which inhibits its cellular replication [9].

**Figure 5:**
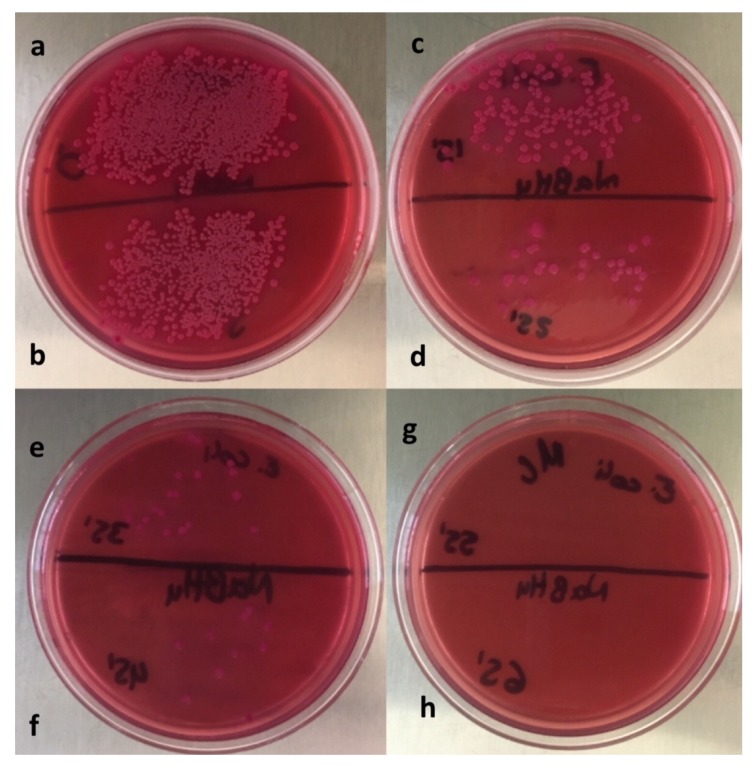
Photograph of plate with *Escherichia coli* and MacConkey as agar incubated with 5% Ag/Bovine bone powder at different times. Minimum inhibitory concentration (MIC) of 7.5 µg/mL (**a**) sample without support, (**b**) 5, (**c**) 15, (**d**) 25, (**e**) 35, (**f**) 45, (**g**) 55, and (**h**) 65 min after adding the Ag/bone system.

We tried to use the turbidimetry method, which was not possible because the synthesized material not only has a bactericidal effect, but also contributed to reducing the turbidity of the medium, and acted in a very fast way, preventing the use of this method. This could be confirmed with the sowing after the use of the support, where the powder with 1% of silver considerably inhibits the growth. The better elimination yield of bacteria with the 5% Ag/Bovine bone powder system is attributed to the higher amount of silver NPs found on the support and therefore a higher amount of active sites to which the bacteria can be attached. Based on these results, the 5% Ag nanocomposite was chosen to establish the time when the antimicrobial effect was total (see Figure 5).

Minimum inhibitory concentration for the system Ag/bovine bone powder and *Escherichia coli* was found to be 7.5 µg/mL. This concentration is lower than that reported by other research groups [31,39,40,41] when using other type of supports. Therefore, it is shown here a technical advantage of controlling the size of NPs. Besides, the synthesized biocomposite can be retrieved and used in other alike processes, i.e., where a material with bactericide effect is needed.

**Figure 6:**
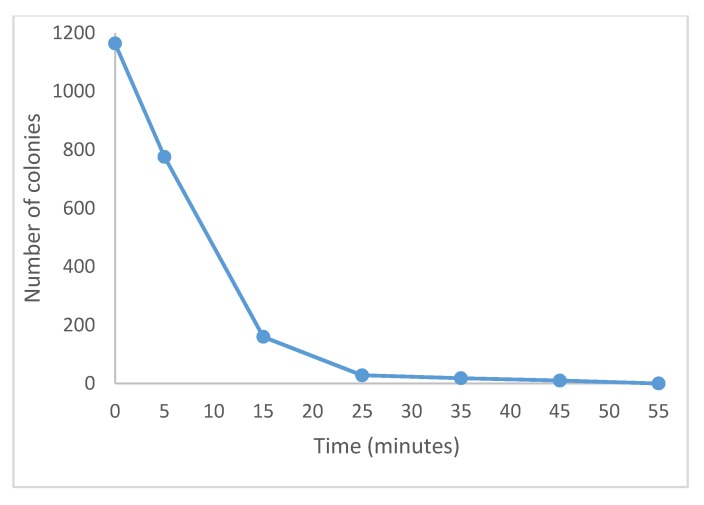
Biocomposite effect (5% Ag) on *Escherichia coli* colonies number at different times.

Figure 6 depicts the number of colonies versus contact time with the biocomposite. It can be observed that the bactericide effect was total after 46.8 min. This time is noticeably lower than that reported by other researchers [31,41,42].

**Figure 7:**
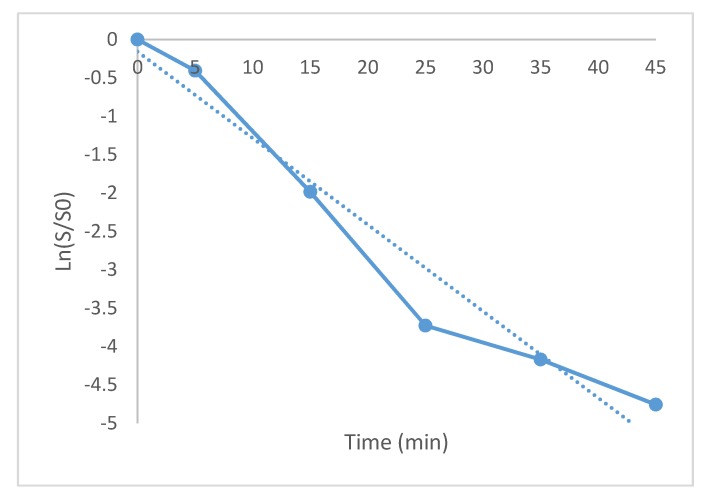
Graph to calculate the kinetic decay constant of *Escherichia coli* with 5% Ag/bovine powder. Continuous line: Experiments, dotted line: linear regression.

In order to establish the kinetic decay constant, a linear regression was established by plotting the ln (S/So) versus time (Figure 7), where S_0_ was the initial number of microorganisms, S was the number of surviving microorganisms at time t. The slope of such line was found to be 0.1128 min^−1^ and the regression coefficient was 0.95.

The decimal reduction time (DRT) was found to be 20.39 min. This is the required time to decrease 90% of bacteria in a logarithm cycle and it was calculated by means of DRT = 2.3/k.

It has been suggested [9,10,41] that the inhibitory action mechanism exerted by silver ions can be ascribed to silver (+) ions being electronegatively attracted to the sulfate and phosphate (−) groups of the bacteria cell membrane. This attraction interferes with their primary properties, such as permeability and respiration; hampering its capacity for replication and leading to the denaturation of proteins.

The methodology used in this research was without stirring, which is expected to make the bacteria decay faster.

## 4. Conclusions

An environmentally friendly bactericide system was prepared. This system consists of silver nanoparticles on bovine powder. The nanoparticles support is low-cost, biodegradable, and the treatment is relatively easy. Bone hydroxy and phosphates groups with negative charge exerted an electrostatic attraction to positive charges of silver nanoparticles, thus retaining them onto its surface. By the reduction of metallic salts, using NaBH_4_, the size of silver nanoparticles in the 5% silver/bovine bone powder was 10.5 ± 3.3 nm, with mainly quasi-spherical shape and with good polydispersity. Ag/bovine powder systems were synthesized with two contents, 1% and 5% weight, the last one exhibiting a stronger bactericide effect for *Escherichia coli* after 46.8 min without stirring. The system with 1% only managed to inhibit the growth but did not completely eliminate the bacteria. Minimal inhibitory concentration was found to be 7.5 µg/mL.

## Supplementary Materials

The following are available online at https://www.mdpi.com/1996-1944/13/2/462/s1. Figure S1: ImageJ software screen example to conduct the measurement of silver particles and establish the particle size distribution. Figure S2: Examples of TEM images used to establish the particle size distribution in the 5% Ag/bovine powder system.
