# Supplementary material for: Bionanotechnology: Silver Nanoparticles Supported on Bovine Bone Powder Used as Bactericide
Source: Materials (Basel). 2020 Jan 18;13(2):462. doi: 10.3390/ma13020462 (PMC7014155; doi:10.3390/ma13020462)
Supplement: Supplementary file 1 [file materials-13-00462-s001.pdf]

materials

Supplementary Materials: Bionanotechnology: Silver nanoparticles supported on bovine bone powder used as bactericide

Sergio A. Gama Lara *, Martha Stephanie Pérez Mendoza, Rafael Alberto Vilchis-Nestor and Reyna Natividad *

In order to establish the particle size distribution, about 30 TEM images were analysed in an open access software called ImageJ developed by the National Institutes of Health (NIH) from USA. An image example of the software is given in figure S1. With this software, 402 Ag particles were measured for the 1% Ag/bone system and 404 for the 5% Ag/bone material.

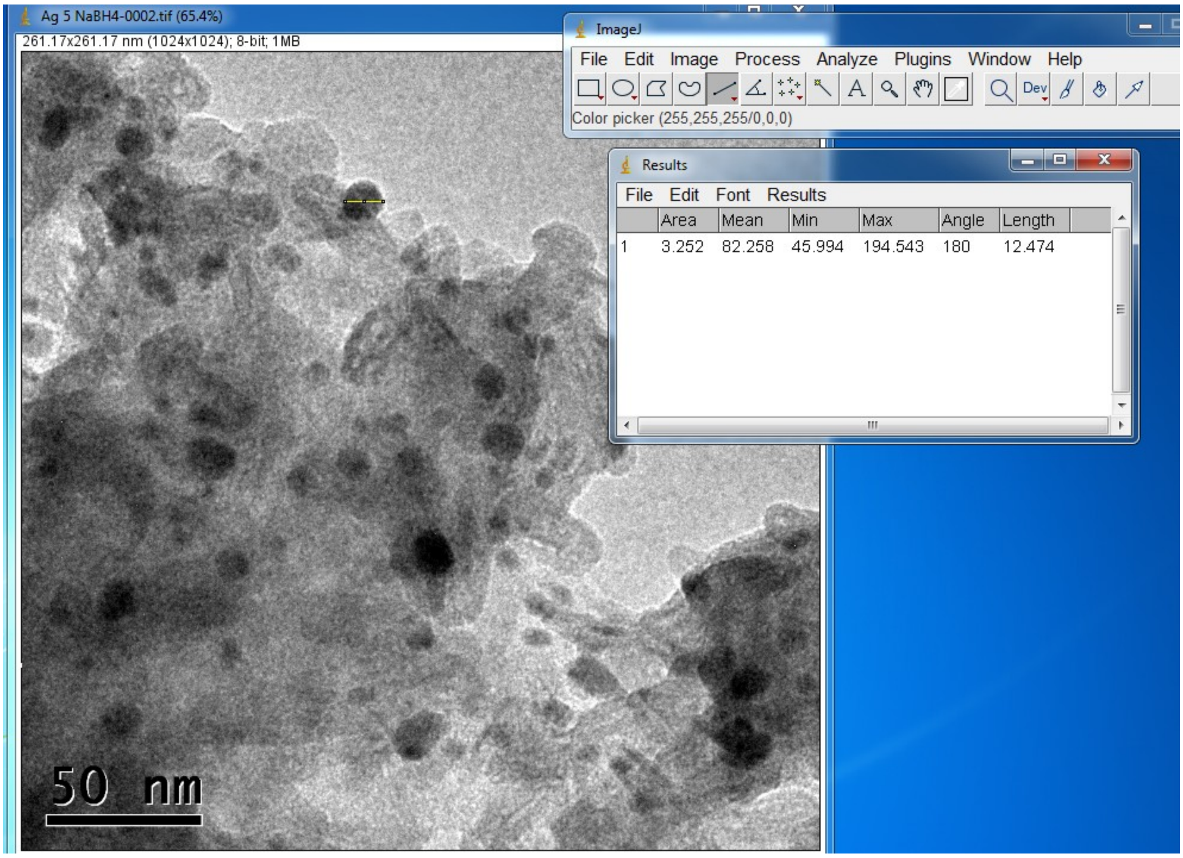

Figure S1. ImageJ software screen example to conduct the measurement of silver particles and establish the particle size distribution.

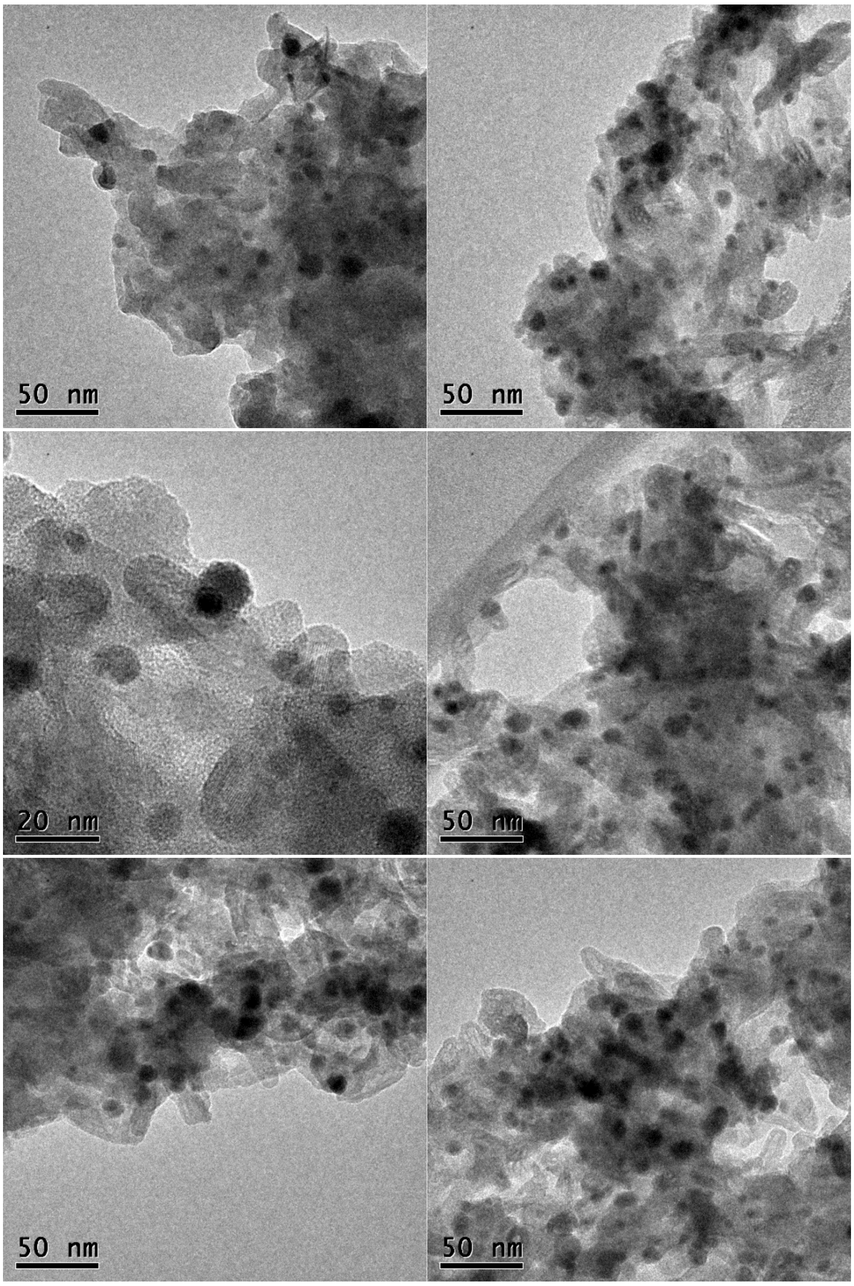

Figure S2. Examples of TEM images used to establish the particle size distribution in the 5% Ag/bovine powder system.
